# Family perspectives of COVID-19 research

**DOI:** 10.1186/s40900-020-00242-1

**Published:** 2020-11-30

**Authors:** Shelley M. Vanderhout, Catherine S. Birken, Peter Wong, Sarah Kelleher, Shannon Weir, Jonathon L. Maguire

**Affiliations:** 1 Li Ka Shing Knowledge Institute of St. Michael’s Hospital, Unity Health Toronto, 209 Victoria St, Toronto, ON M5B 1T8 Canada; 2 Division of Paediatric Medicine and the Paediatric Outcomes Research Team, The Hospital for Sick Children, 555 University Avenue, Toronto, Ontario M5G 1X8 Canada; 3 Child Health Evaluative Sciences, The Hospital for Sick Children, Peter Gilgan Centre for Research & Learning, 686 Bay Street, 11th floor, Toronto, Ontario M5G 0A4 Canada; 4 Patient partners, Toronto, Canada; 5 Toronto, Canada

**Keywords:** COVID-19, Children, Families

## Abstract

**Background:**

The COVID-19 pandemic has uniquely affected children and families by disrupting routines, changing relationships and roles, and altering usual child care, school and recreational activities. Understanding the way families experience these changes from parents’ perspectives may help to guide research on the effects of COVID-19 among children.

**Main body:**

As a multidisciplinary team of child health researchers, we assembled a group of nine parents to identify concerns, raise questions, and voice perspectives to inform COVID-19 research for children and families. Parents provided a range of insightful perspectives, ideas for research questions, and reflections on their experiences during the pandemic.

**Conclusion:**

Including parents as partners in early stages of COVID-19 research helped determine priorities, led to more feasible data collection methods, and hopefully has improved the relevance, applicability and value of research findings to parents and children.

## Plain English summary

Understanding the physical, mental, and emotional impacts of the COVID-19 pandemic for children and families will help to guide approaches to support families and children during the pandemic and after. As a team of child health researchers in Toronto, Canada, we assembled a group of parents and clinician researchers during the COVID-19 pandemic to identify concerns, raise questions, and voice perspectives to inform COVID-19 research for children and families. Parents were eager to share their experience of shifting roles, priorities, and routines during the pandemic, and were instrumental in guiding research priorities and methods to understand of the effects of COVID-19 on families. First-hand experience that parents have in navigating the COVID-19 pandemic with their families contributed to collaborative relationships between researchers and research participants, helped orient research about COVID-19 in children around family priorities, and offered valuable perspectives for the development of guidelines for safe return to school and childcare. Partnerships between researchers and families in designing and delivering COVID-19 research may lead to a better understanding of how health research can best support children and their families during the COVID-19 pandemic.

## Background

Children and families have been uniquely affected by the COVID-19 pandemic. While children appear to experience milder symptoms from COVID-19 infection than older individuals [1], sudden changes in routines, resources, and relationships as a result of restrictions on physical interaction have resulted in major impacts on families with young children. In the absence of school, child care, extra-curricular activities and family gatherings, children’s social and support networks have been broadly disrupted. Stress from COVID-19 has been compounded by additional responsibilities for parents as they adapt to their new roles as educators and playmates while balancing full-time caregiving with their own stressful changes to work, financial and social situations. On the contrary, families with greater parental support and perceived control have had less perceived stress during COVID-19 [2].

The COVID-19 pandemic has rapidly sparked research activity across the globe. Patient and family voices are increasingly considered essential to research agenda and priority setting [3]. Understanding the physical, mental, and emotional consequences of the COVID-19 pandemic for families will inform approaches to support parents and children during the pandemic and after. In this unusual time, patient and family voices can be valuable in informing health research priorities, study designs, implementation plans and knowledge translation strategies that directly affect them [4].

### Setting

As a multidisciplinary team of child health researchers with expertise in general paediatrics, nutrition and mental health, we assembled a group of nine parents to identify concerns, raise questions, and voice perspectives to inform COVID-19 research for children and families. Parents were recruited from the TARGet Kids! primary care research network [5], which is a collaboration between applied health researchers at the SickKids and St. Michael’s Hospitals, primary care providers from the Departments of Pediatrics and Family and Community Medicine at the University of Toronto, and families. Parents were contacted by email and invited to voluntary meetings on April 7 and 23, 2020 via Zoom [6] for 3 h. In an unstructured discussion, we asked how parents imagined research about COVID-19 could make an impact on child and family well-being. Parents were encouraged to share their lived experience and perspectives on the anticipated effects of COVID-19 and social distancing policies on their children and families, and opinions to inform how research on child mental and physical health during and after the pandemic could best be conducted. Parents had opportunities to review proposed data collection tools such as smartphone apps and serology testing devices, and provided feedback about the feasibility and meaningfulness of each. Content, frequency and organization of questionnaires were also reviewed by parents to ensure they were appropriate in length and feasible to complete.

### Parent perspectives

Parents were optimistic that research would provide an understanding of the effects of COVID-19 on families and deliver solutions to minimize negative effects and bolster positive effects. Parents wondered about several questions which they hoped research would answer including: What will be the effects of physical distancing and disrupted routines for my children? How can I help my children develop healthy coping habits? How can I appropriately talk about the virus with my children? What factors might predict resiliency against negative effects of the pandemic among children and families, and how can these be strengthened?

Parents speculated what risks children might face as a result of schoolwork transitioning to home, educational activities provided online, child care being limited or unavailable, social relationships changing, sports and extra-curricular activities being cancelled, and stress and anxiety increasing at home. Some parents reflected on feeling some relief from not having to coordinate usual extracurricular activities. However, they expressed frustration in finding high quality educational activities and resources to support physical and mental health for their children during physical isolation. Parents voiced a need for a centralized, accessible hub with peer reviewed, high quality resources to keep children entertained and supported while spending more time indoors, away from usual activities and school. They hoped for resources to help families adjust to new routines and roles, as well as answer children’s questions in truthful ways that would not increase anxiety.

Parents were curious about studying the impact of COVID-19 on children and families. How would researchers use information about children who are affected physically, mentally, or socially by the pandemic? What could be the possible implications of testing for COVID-19 on social relationships and parents’ employment? This question generated discussion about difficult positions families of lower socio-economic status, who may need to maintain attendance at work but have a suspected COVID-19 infected household member. Would health and social care for children going forward reflect the unique ways they had been impacted by changes in their daily routines and relationships? How can families return to school and everyday routines with a minimum of disruption? What will be done to prepare children and families for emergency situations in the future? Considering these questions may lead child health researchers to study relevant and contemporary concepts to families during the COVID-19 pandemic.

When presented with options to include more measures on other family members, parents maintained that the focus of our COVID-19 research should be on children. Parents provided essential feedback about the length and frequency of questionnaires, to ensure they were appropriate given the limited time available for completing them. Parent involvement early in the research process helped to direct research priorities, informed data collection strategies and hopefully has increased the relevance of research conducted for children and families. Conducting a follow-up meeting with parents was important to understand shifting concerns and ensure data collection was reflecting current routines, habits and policies affecting families.

## Conclusions

As researchers who are seeking to understand the impact of COVID-19 on children and families, we felt it important to involve families in designing and implementing new research. First-hand experience that parents have in navigating the COVID-19 pandemic with their children contributed to co-building between researchers and research participants. Parents were generous with their time and provided insightful, honest suggestions for how researchers could create knowledge that would be directly relevant to them. Next steps will include expanding our dialogue with a more diverse group of parents in terms of gender, as all parents in our meetings were women, and ethnicity to better represent the diversity of Toronto. Other researchers conducting COVID-19 research among children and families may consider engaging parents and caregivers in preliminary stages to identify priorities, understand lived experiences and help guide all stages of the research process. This presents value in focusing research on the most important priorities for families and developing data collection methods which are feasible in the context of the COVID-19 pandemic. As the nature of the COVID-19 pandemic is dynamic, ongoing communication between researchers and parents to understand changing perspectives and concerns is important to respond to family needs. We hope that ongoing partnerships between parents and researchers will promote leadership among parents as co-investigators in COVID-19 research, and result in research which addresses the needs of parents and children during the COVID-19 pandemic. Ideally, engaging with families in COVID-19 research will result in findings that will be valuable to families, assist them in developing collective resilience, and provide a foundation for family-oriented research throughout the COVD-19 pandemic and beyond.

## Data Availability

Not applicable.
